# Mutation of the Atypical Kinase ABC1K3 Partially Rescues the PROTON GRADIENT REGULATION 6 Phenotype in *Arabidopsis thaliana*

**DOI:** 10.3389/fpls.2020.00337

**Published:** 2020-03-25

**Authors:** Thibaut Pralon, Joy Collombat, Rosa Pipitone, Brigitte Ksas, Venkatasalam Shanmugabalaji, Michel Havaux, Giovanni Finazzi, Paolo Longoni, Felix Kessler

**Affiliations:** ^1^Laboratory of Plant Physiology, Institute Biology, University of Neuchâtel, Neuchâtel, Switzerland; ^2^Aix Marseille University, Centre National de la Recherche Scientifique (CNRS), Commissariat à l’Énergie Atomique et aux Énergies Alternatives (CEA), UMR 7265, Biosciences et Biotechnologies Institute of Aix-Marseille, Saint-Paul-lez-Durance, France; ^3^Université Grenoble Alpes, Centre National de la Recherche Scientifique (CNRS), Commissariat à l’Énergie Atomique et aux Énergies Alternatives (CEA), Institut National de la Recherche Agromique (INRA), Interdisciplinary Research Institute of Grenoble - Cell and Plant Physiology Laboratory (IRIG-LPCV), Grenoble, France

**Keywords:** photosynthetic electron transport, plastoquinone pool, plastoglobule, high light acclimation, NPQ

## Abstract

Photosynthesis is an essential pathway providing the chemical energy and reducing equivalents that sustain higher plant metabolism. It relies on sunlight, which is an inconstant source of energy that fluctuates in both intensity and spectrum. The fine and rapid tuning of the photosynthetic apparatus is essential to cope with changing light conditions and increase plant fitness. Recently PROTON GRADIENT REGULATION 6 (PGR6-ABC1K1), an atypical plastoglobule-associated kinase, was shown to regulate a new mechanism of light response by controlling the homeostasis of photoactive plastoquinone (PQ). PQ is a crucial electron carrier existing as a free neutral lipid in the photosynthetic thylakoid membrane. Perturbed homeostasis of PQ impairs photosynthesis and plant acclimation to high light. Here we show that a homologous kinase, ABC1K3, which like PGR6-ABC1K1 is associated with plastoglobules, also contributes to the homeostasis of the photoactive PQ pool. Contrary to PGR6-ABC1K1, ABC1K3 disfavors PQ availability for photosynthetic electron transport. In fact, in the *abc1k1/abc1k3* double mutant the *pgr6*(*abc1k1*) the photosynthetic defect seen in the *abc1k1* mutant is mitigated. However, the PQ concentration in the photoactive pool of the double mutant is comparable to that of *abc1k1* mutant. An increase of the PQ mobility, inferred from the kinetics of its oxidation in dark, contributes to the mitigation of the *pgr6*(*abc1k1*) photosynthetic defect. Our results also demonstrate that ABC1K3 contributes to the regulation of other mechanisms involved in the adaptation of the photosynthetic apparatus to changes in light quality and intensity such as the induction of thermal dissipation and state transitions. Overall, we suggests that, besides the absolute concentration of PQ, its mobility and exchange between storage and active pools are critical for light acclimation in plants.

## Introduction

The photosynthetic conversion of light energy into chemical energy occurs via a series of redox reactions resulting in electron transport along the thylakoid membrane. The linear electron transport begins with water splitting at the level of photosystem II (PSII) and ends at photosystem I (PSI) with the reduction of NADP^+^ by ferredoxin. Both PSII and PSI utilize photonic energy to fuel the redox reactions. Electrons are transferred from PSII to PSI via the cytochrome *b6f* complex (cyt *b6f*). At the QB site of PSII, a molecule of plastoquinone (PQ) is reduced twice and protonated to form plastoquinol (PQH_2_). The PQH_2_ can then diffuse within the thylakoid membrane to reach the QO site of cyt *b6f*. Oxidation of PQH_2_ at cyt *b6f* occurs through the Q-cycle that releases protons into the thylakoid lumen contributing to the formation of the trans-thylakoid proton gradient. In addition, two electrons are released, one of which returns to PQ pool, while the other is transferred via the plastocyanin to PSI (Tikhonov, 2014). The proportion of PQ that participates in electron transport in the thylakoid membrane is considered as the photoactive PQ pool; whereas the remaining proportion, which is approximately 60–70% of the total PQ, constitutes the non-photoactive pool and is largely stored inside thylakoid-associated lipid droplets known as plastoglobules (PG) (Kruk and Karpinski, 2006; Block et al., 2013; Ksas et al., 2018).

To shuttle electrons, PQ has to rapidly navigate in the thylakoid lipid bilayer (Blackwell et al., 1994). However, the thylakoid membrane is crowded with integral proteins, covering up to 70% of the surface in the grana stacks, which drastically restrict PQ diffusion, especially at the long-range (Kirchhoff et al., 2000). Thylakoid membranes are close to the percolation threshold, therefore it has been suggested that the organization of the protein supercomplexes creates lipid microdomains that facilitate PQ mobility and thus electron shuttling between PSII and cyt *b6f* (Lavergne and Joliot, 1991; Kirchhoff et al., 2000; Kirchhoff, 2014). On the other hand, long-range mobility of the PQ is also important, for instance to mobilize the non-photoactive pool when damaged PQ molecules have to be replaced as it was proposed to occur during high light stress (Ksas et al., 2018). Therefore, the mobility of plastoquinone/ol molecules within the thylakoid lipid bilayer is a critical parameter to ensure electron transport and maintain the photosynthetic electron transport chain (ETC).

Apart from the role of PQ as electron carrier, its redox state is an important signal in the regulation of many physiological processes within the chloroplast such as state transitions (as described below), gene expression, carotenoid biosynthesis, and antioxidant activity (Karpinski et al., 1999; Li et al., 2009; Suzuki et al., 2012). A rapid readout of the PQ redox state allows photosynthetic adaptation to changes in environmental conditions and therefore increases plant fitness in the natural environment. The adaptation to such varying light conditions is essential for plants to maintain the highest photosynthetic efficiency while avoiding photo-induced damage. To alleviate the negative effects of an imbalance between the activity of the two photosystems, plants have developed a short-term adaptive mechanism: the state transitions. This process allows the re-equilibration of the light energy input between the two photosystems on a time scale of a few minutes by re-allocating part of the mobile light-harvesting complexes II (LHCII) (Allen et al., 1981; Rochaix, 2007, 2013). Phosphorylation of LHCII by the STN7 kinase, the activity of which is dependent on the PQ redox state, allows its movement from PSII to PSI (state 2) (Bellafiore et al., 2005; Shapiguzov et al., 2016; Dumas et al., 2017). The process is reverted by the dephosphorylation of LHCII, operated by the PPH1/TAP38 phosphatase (state 1) (Pesaresi et al., 2010, 2011; Shapiguzov et al., 2010; Trotta et al., 2016). To prevent harmful effects of excess light, plants can dissipate the energy excess as heat by a set of regulated mechanisms summarily referred to as non-photochemical quenching (NPQ). These mechanisms include the rapid rearrangement to a “quenched” state of the LHCII antenna dependent on the PSBS protein and the conversion of the xanthophylls associated to LHCII from violaxanthin to zeaxanthin. The sum of all components creates a system with differential kinetics activated within a few seconds to hours (Dall’Osto et al., 2005; Joliot and Finazzi, 2010; Nilkens et al., 2010; Sylak-Glassman et al., 2014; Ruban, 2016, 2018). Nonetheless, upon continuous light stress, ROS can be produced at the PSII reaction center leading to loss of the PSII activity by damaging the core protein D1 (PsbA). Loss of PSII activity, known as photoinhibition (qI), contributes to the dissipation of excess light (Gong and Ohad, 1991; Miyao, 1994; Vasilikiotis and Melis, 1994). To restore PSII activity after inhibition there is an efficient repair cycle (Aro et al., 1993; Rintamaki et al., 1996, 1997; Theis and Schroda, 2016). The replacement of damaged D1 (PsbA) is facilitated by the phosphorylation of the core proteins of the PSII reaction center by STN8 kinase and requires PSII to migrate from the grana to the stroma lamellae (Aro et al., 1993; Bonardi et al., 2005; Tikkanen et al., 2008; Theis and Schroda, 2016; Li et al., 2018). Photo oxidative stress also triggers other responses allowing the chloroplast to alleviate the damage. These responses involve regulation of gene expression (Pfannschmidt et al., 1999; Pfannschmidt, 2003), structural changes of the thylakoids (Moejes et al., 2017) and synthesis of antioxidant molecules (Munné-Bosch and Alegre, 2002; Kanwischer et al., 2005; Gruszka et al., 2008; Nowicka and Kruk, 2012; Block et al., 2013; Martinis et al., 2014; Ksas et al., 2015, 2018; Spicher et al., 2016; Ferretti et al., 2018).

An important player in the plant stress response is the plastoglobule (PG). PGs are small lipid droplets, attached to the outer lipid leaflet of the thylakoid membrane, delimited by a membrane lipid monolayer consisting mostly of galactolipids and coated with proteins (Austin et al., 2006). Several neutral lipids including prenylquinones, carotenoids, triacylglycerols, phytolesters fill the plastoglobule (Lichtenthaler and Peveling, 1966; Steinmuller and Tevini, 1985; Gaude et al., 2007; Zbierzak et al., 2010; Lippold et al., 2012; Lundquist et al., 2013; Rottet et al., 2016; van Wijk and Kessler, 2017). In response to various stresses PGs increase in size and number (Taylor and Craig, 1971; Hall et al., 1972; Gaude et al., 2007; Martinis et al., 2014; Zechmann, 2019). Physical connections between PG and the thylakoid membranes suggest bidirectional lipid trafficking between these two compartments (Austin et al., 2006). Besides being a lipid storage site, the PG proteome revealed the presence of specific proteins, several of which are involved in prenylquinone metabolism (Vidi et al., 2006; Ytterberg et al., 2006; Lundquist et al., 2012).

After fibrillins (Lundquist et al., 2012), the second most abundant protein family in PG is composed of homologs of the ABC1 (Activity of BC1 complex) atypical kinases (Lundquist et al., 2012). The ABC1 domain has been conserved through evolution, suggesting that it has a crucial role (Lohscheider and Rio Bartulos, 2016). In microorganisms as well as in human cells, ABC1 proteins were shown to be essential in ubiquinone synthesis and in mitochondrial electron transport (Bousquet et al., 1991; Brasseur et al., 1997; Poon et al., 2000; Mollet et al., 2008). Six members of the ABC1-like kinase family are found in the PG proteome. A member of this family, ABC1K1 (At4g31390), was identified as *PGR6* in a genetic screen to identify mutants affected in proton gradient formation (PGR) (Shikanai et al., 1999). PGR mutants are characterized by high chlorophyll fluorescence and reduced NPQ under different light conditions (from 50 to 500 μmol of photons m^–2^ s^–1^) (Shikanai et al., 1999; Martinis et al., 2014; Yamori and Shikanai, 2016). In the *abc1k1* mutant, the electron transport rate as well as NPQ are constitutively limited in a light fluency dependent manner when compared to the wild type. Under prolonged exposure to high light, *abc1k1* adult plants exhibited almost complete photoinhibition during the early days of treatment and after several days PSII maximum efficiency recovered despite the plants still being still exposed to high light (Martinis et al., 2014). Nonetheless, the metabolic profile of *abc1k1* was profoundly altered. In particular, plants displayed a decrease in tocopherol accumulation and a shift from starch production to soluble sugars (Martinis et al., 2014).

ABC1K1 was identified as BDR1 (Bleached dwarf under red light) (Huang et al., 2015; Yang et al., 2016). During early seedling development under continuous red light, the mutant is severely stunted and has white cotyledons. Since the bleaching phenotype was accompanied by a specific diminishment of the photosystem D1 (PsbA) protein but not that of other photosynthetic proteins tested [D2 (PsbD); PsbC; PsbB] the pale phenotype was attributed to photobleaching (Huang et al., 2015; Yang et al., 2016). A repressor of the *bdr1* mutation, RBD1 (repressor of *bdr1*) was also identified as the ABC1K1 homolog ABC1K3. As the *abc1k3* mutation repressed the *bdr1*(*abc1k1*) phenotype, it led to the hypothesis that the two homologs have opposing functions (Huang et al., 2015). Furthermore, *abc1k3* adult plants are not severely affected by prolonged high light and they showed a decreased plastochromanol accumulation (Martinis et al., 2013). However, previous investigations on adult plants reported that the double *abc1k1/abc1k3* mutation results in an additive, senescence-like phenotype characterized by conditional degreening, including the loss of chlorophyll and photosystem proteins, and recruitment of the jasmonate pathway to PG under prolonged high light treatment (Lundquist et al., 2013). Furthermore, ABC1K1 and ABC1K3 may interact and form a complex that may be involved in the stabilization of plastoglobule proteins (Lundquist et al., 2013; Martinis et al., 2013).

Recently, a molecular mechanism explaining the *pgr6*(*abc1k1*) defect was proposed: ABC1K1 would be required for the homeostasis of photoactive PQ. Indeed, upon high light, the photoactive PQ pool in *abc1k1* mutant becomes limiting and this can explain the diminished linear electron transport and NPQ, the dephosphorylation of the LHCII antenna and perturbation of the state transitions; all these leading to an overall decrease in the photosynthetic efficiency (Pralon et al., 2019).

In this study, we investigated the impact of the *abc1k3* mutation in the *pgr6(abc1k1)* mutant background. In particular we focused on the capacity of a double mutant, lacking both *ABC1K1* and *ABC1K3*, to acclimate to a short high light treatment (3 h, 500 μmol⋅m^–2^⋅s^–1^). We found that the *abc1k3* mutant has no photosynthetic defect compared to the wild type under the tested conditions. However, by stacking this mutation with *abc1k1* we observe a partial alleviation of all the photosynthetic defects previously reported in *abc1k1* (Shikanai et al., 1999; Martinis et al., 2014; Pralon et al., 2019). Surprisingly, the phenotype complementation does not originate from an effect on the size of the photoactive PQ pool but rather from an effect on PQ mobility in the thylakoid membrane. This evidence suggests that there is a push-pull relationship of ABC1K1 and ABC1K3 with regard to the mobility of PQ, and that this regulated process is fundamental to ensure the photosynthetic efficiency under high light.

## Materials and Methods

### Plants Material and Treatments

The wild type *Arabidopsis thaliana* refers to var. Columbia-0 (Col-0). *abc1k1.1* (Salk_068628), *abc1k1.2* (Salk_130499C) *abc1k3.1* (Salk_128696), or with *abc1k3.2* (Sail_918_E10) T-DNA insertion lines were purchased at Nottingham Arabidopsis Stock Centre (NASC)^1^. The double mutant lines *abc1k1/akc1k3.1* and *abc1k1/akc1k3.2* were produced by crossing *abc1k1.1* mutant with *abc1k3.1* and *abc1k3.2*, respectively. TDNA insertion was verified by PCR, and primers were listed in Supplementary Table 1. The mutant lines *stn7/stn8* (Fristedt et al., 2009) and *sps2* (Block et al., 2013) were kindly provided by the respective research groups. The *ptox* mutant was obtained from NASC, and corresponds to the previously characterized immutans variegation mutant (Wetzel et al., 1994).

Plants were grown in pots on soil pre-treated with solbac (Andermatt) with standard light conditions (120 μmol m^–2^ s^–1^, 8 h light/16 h dark) in a controlled environment room maintaining a daily temperature of 22 ± 1°C. For high light treatment, 4–5 weeks old plants were exposed for 3 h to 500 μmol m^–2^ s^–1^ of white light (FutureLED), always at 22 ± 1°C.

Leaf samples were collected directly under the light in the growth chamber, and immediately snap frozen in liquid nitrogen, and stored at −20°C.

### Photosynthetic Parameters

Before measuring the photosynthetic performance, the plants were kept in the dark for at least 10 min. The measurements were performed using a Fluorcam (Photon System Instrument, Czech Republic)^2^ with a modified light curve protocol. Briefly, after measuring the minimal fluorescence (F_0_) and the maximal fluorescence during a saturating pulse (F_M_) in dark, the plants were exposed to increasing blue light (470 nm) intensities for 1 min following this scheme: 2.5–95–347–610–876–1145 μmol m^–2^ s^–1^. At the end of every light period, a saturating flash was used to measure the maximal fluorescence in light (F_M_’). The fluorescence recorded before the peak were used as F_S_ (steady-state chlorophyll fluorescence in the light). Three parameters were calculated from the above values: Maximum quantum yield of photosystem II Φ_MAX_ = (F_V_/F_M_); quantum yield of photosystem II Φ_PSII_ = (F_M_’ – F_S_)/F_M_’ and Non-Photochemical Quenching NPQ = (F_M_ – F_M_’)/F_M_’. To assess the extent of the “state transitions” the plants were exposed to 10 min red light (50 μmol m^–2^ s^–1^ 660 nm peak measured as PPFD) supplemented with far-red (17 μmol m^–2^ s^–1^ calculated from the 733 nm peak area considering values between 500 and 800 nm) followed by 10 min of the red light only. The maximal fluorescence in the light was measured at the end of both 10 min periods to obtain F_M_ST1 when far-red was supplemented and “state I” promoted and F_M_ST2 after 10 min of pure red, which promotes the transition toward “state II.” The extent of the quenching related to state transition (qT) was calculated as qT = (F_M_ST1 – F_M_ST2)/F_M_.

### P_700_ Oxidation

The photooxidation of the photosystem I reaction center (P_700_) was assessed by the increase in absorption at 810 nm after deconvolution of the plastocyanin absorption as previously described (Joliot and Joliot, 2006). The measurement was performed with a JTS-10 LED spectrometer (BioLogic Science Instruments) in absorbance mode equipped with a Far-red (FAR) LED peaking at 735 nm filtered through three Wratten filters 55 to block the wavelengths shorter than 700 nm, a red LED, peaking at 640 nm, was used for the actinic light. To estimate maximum extent of P_700_^+^ a saturating white light flash was superimposed on the top of the FAR. The measurement of the maximum number of electrons contained in the electron transport chain (ETC) per PSI was performed as follows. The plants were pre-incubated 2 min under a strong white light (500 μmol m^–2^ s^–1^) to activate CO_2_ assimilation in the leaves and thus reduce the contribution of the cyclic electron flow (Joliot and Joliot, 2006), which can only be reactivated after prolonged dark incubation (Joliot and Joliot, 2006; Trouillard et al., 2012). This allows only electrons from the linear ETC, including the photoactive PQ pool, to be available for PSI reduction. Detached leaves were put inside the holder and subjected to 2 min of FAR to oxidize the ETC followed by 2 s of dark allowing the reduction of P_700_. The kinetic of the subsequent reoxidation induced by FAR were followed with a saturating flash of actinic light (1000 μmol m^–2^ s^–1^ for 100 μs) to fully reduce the ETC or without so that the ETC was fully oxidized. The ratio between the lag times between the FAR onset and the beginning of the P_700_ oxidation in these two conditions is used as a proxy for the number of available electrons per PSI (Pralon et al., 2019).

### Chlorophyll *a* Fluorescence Curve Kinetics (OJIP, JIP-Test)

Plants were dark-adapted for at least 10 min before measurements and then a single leaf detached in dark and put in a clip holder for the Plant Efficiency Analyzer (M-PEA 2; Hansatech Ltd.). The chlorophyll *a* fluorescence induction curve was recorded and the JIP-parameters recovered with M-PEA software Hansatech Ltd. The measurement was automatically repeated after increasing intervals of dark (4, 8, 12, 16, 20, 24 s) and far-red light (0.05, 0.1, 0.2, 0.4, 0.8, 1.6 s). The parameters extracted from the first pulse after the dark incubation were used to assess the steady state of the ETC after moderate light or 3 h of high light (Stirbet et al., 2018). The yield of the electron transport from Q_A_ to Q_B_ (ΦET2o) and the yield of the transport to final PSI acceptors (ΦRE1o) were calculated according to the JIP-Model described in Strasser et al. (2010) and Kalaji et al. (2014a, b). The variable fluorescence at 3 ms (V_J_ = F_J_/F_M_) value was used as a proxy of the redox state of the photoactive plastoquinone (Tóth et al., 2007). The data points of V_J_ over time were interpolated with a logarithmic function with RStudio (RStudio, Inc.).

### Plastoquinone Analysis

The analysis of the photoactive PQ pool, and of the total pool were performed as previously described (Pralon et al., 2019). Briefly, leaf disks of 0.8 cm of diameter were collected from 5 weeks old plants. The disks were exposed either to 15 s of saturating light (2000 μmol m^–2^ s^–1^) or to 2 min of far-red light (735 nm, 5.5 μmol m^–2^ s^–1^). The samples were flash frozen and used for total lipid analysis as described in Kruk and Karpinski (2006) and Ksas et al. (2015, 2018). The photoactive PQ pool was determined from the difference between the reduced PQ after high light (maximal reduction of the photoactive PQ pool) and the amount measured after far-red light (maximal oxidation of the photoactive PQ pool).

### Immunoblot Analysis

Fully expanded leaves of adult plants (at least 4 weeks old) were collected under the relevant light condition and flash frozen in liquid nitrogen. The leaves were ground to a fine powder in a 1.5 mL microtube with a micro-pestle. Four hundred mircroliter of lysis buffer [100 mM Tris-HCl pH 8.5, 2% SDS, 10 mM NaF, 0.05% of protease inhibitor cocktail for plant (Sigma)] was add to the powder and the material incubated at 37°C for 30 min, debris were removed by centrifugation (5 min at 16,000 g) at room temperature. The chlorophyll concentration of the sample was determined according to Arnon (1949). Proteins were precipitated in chloroform-methanol and the pellet solubilized directly in the gel loading buffer (50 mM Tris-HCl pH 6.8, 100 mM Dithiothreitol, 2% SDS, 0.1% Bromophenol Blue, 10% Glycerol) to a concentration of 0.5 μg chlorophyll/μL. Following denaturation at 65°C for 10 min, 4 μL of each sample were loaded into a 12% Acrylamide SDS gel. After separation by electrophoresis the proteins were transferred to a nitrocellulose membrane. The membrane was decorated using the following antibodies: anti-Actin (Sigma, A 0480) at 1/3000 dilution in 5% fat free milk/PBS, anti-Lhcb2 (Agrisera, AS01 003), anti-D1 (PsbA) (Agrisera, AS05 084), anti-PetC (Agrisera, AS08 330); anti-PsaD (Agrisera, AS09 461), anti-PsaC (Agrisera, AS04 042P), anti-AtpC (Agrisera, AS08 312); at 1/5000 dilution in 5% fat free milk/TBS, and anti-Phosphothreonine (Cell Signaling Technology, #9381) at 1/10,000 in 3% BSA/TBS Tween20 0.1%; anti-PTOX (Agrisera, AS16 3692) at 1/2000 in 6% BSA/TBS Tween20 0.05%. Secondary antibodies (anti-rabbit (Merck, AP132P) or anti-mouse (Sigma, A5278) at 1/3000) conjugated with HRP allow the detection of proteins of interest with 1 mL of enhanced chemiluminescence and 3.3 μL of H_2_O_2_ 3% using an imager for chemiluminescence (Amersham Imager 600, Amersham Biosciences, Inc.).

## Results

### Isolation and Selection of abc1k1/abc1k3 Double Mutants

**FIGURE 1:**
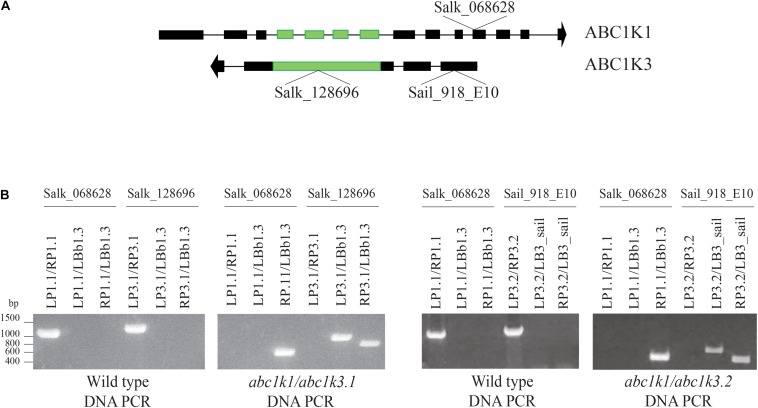
Selection and isolation of *abc1k1/abc1k3* double mutant lines. **(A)** Schematic representation of the position of the T-DNA insertions in *ABC1K1* (At4g31390) and *ABC1K3* (At1g79600) gene sequences. **(B)** Electrophoresis of the amplification products obtained from genomic DNA extracted from Wild type, *abc1k1/abc1k3.1* and *abc1k1/abc1k3.2* confirm the homozygous mutation of both loci. The double mutant *abc1k1/abc1k3.1* was obtained from the crossing between *abc1k1.1* (SALK_068628) and *abc1k3.1* (SALK_128696), while *abc1k1/abc1k3.2* is the result of the crossing between *abc1k1.1* (SALK_068628) and *abc1k3.2* (SAIL_918_E10). The primer used for the amplification are listed in Supplementary Table 1.

ABC1K1 and ABC1K3 are two homologous, atypical kinases located at plastoglobules and share 31.6% identity at the level of the global amino acid sequence (Supplementary Figure 1). To analyze potential interactions between ABC1K1 and ABC1K3 in the regulation of photosynthesis, two double mutant *abc1k1/abc1k3* lines were produced by crossing *abc1k1.1* (Salk_068628) with *abc1k3.1* (Salk_128696) or with *abc1k3.2* (Sail_918_E10). Double *abc1k1/abc1k3* mutant lines were selected and verified by PCR. The genotyping confirmed the presence of TDNA insertions in both genes (Figure 1).

### Thermal Dissipation and Electron Transport Capacities Are Partially Recovered in abc1k1/abc1k3

Independent studies indicate that *abc1k1* is impaired in NPQ as well as electron transport (Shikanai et al., 1999; Martinis et al., 2014; Pralon et al., 2019), while the *abc1k3* mutant did not show defects in those parameters even after prolonged high light exposure (Martinis et al., 2013). To further characterize the *abc1k1/abc1k3* double mutant, we measured photosynthetic parameters such as PSII maximum efficiency [φ_MAX_ (= F_V_/F_M_)], NPQ as well as electron capacity of the ETC in 4–5 weeks old plants grown under moderate light (120 μmol m^–2^ s^–1^) (ML) and after 3 h of high light (500 μmol m^–2^ s^–1^).

**FIGURE 2:**
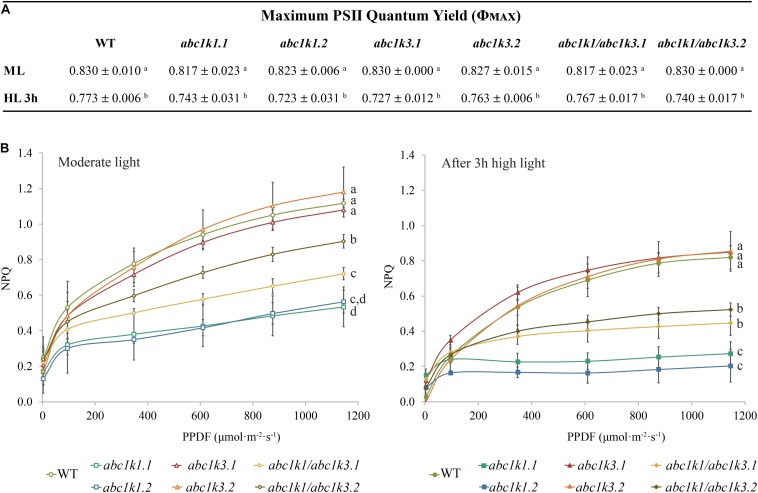
Non-photochemical quenching is partially recovered in *abc1k1/abc1k3* lines compared to *abc1k1*. **(A)** PSII maximum quantum efficiency (ΦPSII = (FM – FO)/FM) and **(B)** non-photochemical quenching (NPQ) of 4–5 weeks old plants of wild type (WT), *abc1k1.1, -2, abc1k3.1, -2* and *abc1k1/abc1k3.1, -2* under moderate light and after 3 h of high light. Plants were dark-adapted 10 min before measurement. Non-photochemical quenching (NPQ = (FM – FM’)/FM’) was calculated from the maximal fluorescence at room temperature recorded after 1 min of exposure at increasing blue light intensities (470 nm). These measures were performed with a Fluorcam (MF800 – PSI). Each value represents the average of a pot containing 2–3 plants. Superscript letters are used to indicate statistically different groups (*p* < 0.05) by paired Student’s *t*-test. Error bars indicate ± SD between different pots (*n* = 3).

The maximum quantum yield of the PSII (φ_MAX_) in wild type (WT), *abc1k1*, *abc1k3*, and *abc1k1/abc1k3* slightly decreased after 3 h of high light but without any significant difference between the lines (Figure 2A). Furthermore, after 3 h of high light, there was no major impact on the PSII yield, and this allowed the measurement of the efficiency of the ETC avoiding potential bias caused by PSII photodamage.

Short-term high light treatment affected NPQ induction, which decreased in all the tested genotypes compared to moderate light conditions. Nonetheless, the NPQ in WT and in *abc1k3* was always higher than the one measured in *abc1k1* lines. Intriguingly, both under moderate light and after high light, the double mutants showed greater NPQ compared to *abc1k1* lines but still less than the WT level (Figure 2B), suggesting a partial rescue of the *pgr6*(*abc1k1*) phenotype in the double mutant.

Starting from P_700_ oxidation kinetics (Joliot and Joliot, 2006) and the JIP-test (Strasser et al., 2010; Kalaji et al., 2014a, b), we evaluated the ETC capacity at the PSI and PSII sides under moderate light and after exposure to high light to determine whether the rescued NPQ in *abc1k1/abc1k3* correlated with an increase in the electron capacity of the ETC.

**FIGURE 3:**
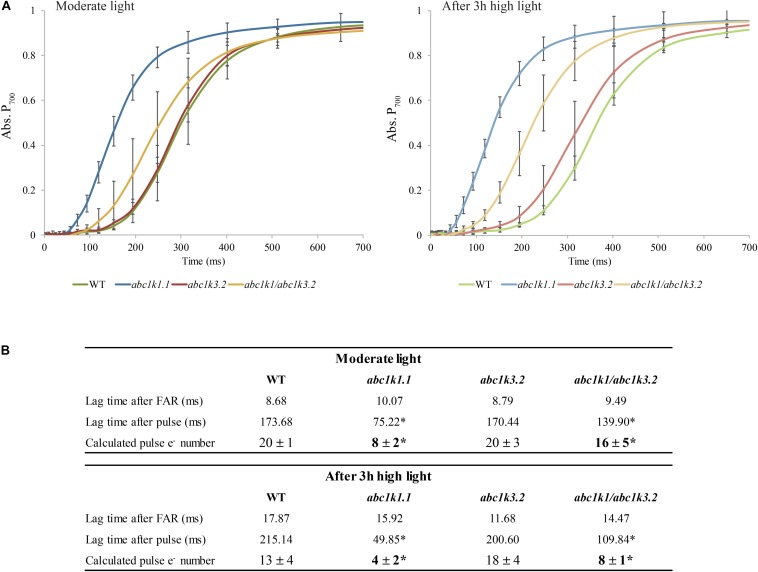
The shortage of electron carriers per photosystem I of *abc1k1* is partially recovered in *abc1k1/abc1k3*. **(A)** Kinetics of P_700_ re-oxidation induced by far-red light were recorded after a saturating light pulse (Time 0 = far-red ON) in wild type, *abc1k1.1, abc1k3.2*, and *abc1k1/abc1k3.2* kept under moderate light and after 3 h of high light. The P_700_ oxidation status was measured by the increase in absorption at 810 nm on fully expanded leaves of each genotype. **(B)** Electron (e^–^) carried by the electron transport chain per photosystem I calculated from the lag time of P_700_ oxidation after a saturating pulse divided by the lag time of the oxidation after dark. Asterisk are used to indicate statistically different groups by Student’s *t*-test (*p* < 0.05) (*n* = 4 biologically independent samples).

P_700_ oxidation kinetics were analyzed after full reduction of the ETC by a saturating flash and its full oxidation by far-red illumination (Figure 3A; Joliot and Joliot, 2006; Pralon et al., 2019). The maximum number of electrons (e^–^) present in the electron transport chain per PSI was assessed from P_700_ oxidation kinetic curves: by dividing the lag time after a strong light pulse (time required to oxidize P_700_ when ETC is fully reduced) by the lag time after far-red exposure (oxidation time of a single electron present in P_700_ reaction center) (Joliot and Joliot, 2006). When sampled under moderate light, *abc1k3* and WT ETC had the same electron capacity per PSI (20 ± 3 and 20 ± 4 electrons respectively), whereas the *abc1k1* ETC carried fewer electrons per PSI (8 ± 1 electrons) (Figure 3B). In the *abc1k1/abc1k3* mutant grown under moderate light the number of electrons carried by the ETC per PSI (16 ± 5) was intermediate between *abc1k1* and the WT. The measure was repeated on plants kept 3 h under high light. After high light treatment, in the WT the number of electrons carried by the ETC per PSI was 13 ± 3, thus it decreased in comparison to the same genotype sampled under moderate light. Similarly, in *abc1k1* as well as *abc1k1/abc1k3* the number of electrons per PSI dropped after high light treatment to 4 ± 2 and 8 ± 1 respectively. The measured electron transport capacity in *abc1k1/abc1k3* was double than that in *abc1k1*, indicating partial rescue of the ETC capacity. The *abc1k3* mutant displayed only a very slight decrease in the number of the ETC carriers per PSI (18 ± 4) upon shifting from moderate light to high light for 3 h, thus more ETC carriers than WT in this condition (Figure 3B).

**FIGURE 4:**
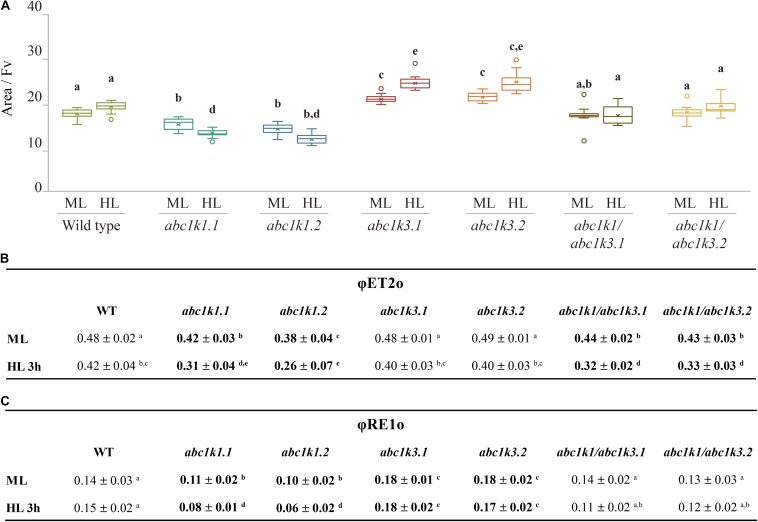
High light as a moderate impact on photosynthetic electron transport fluxes in *abc1k1/abc1k3*. Fully expanded leaves from Wild type (WT), *abc1k1.1, -2, abc1k3.1, -2*, and *abc1k1/abc1k3.1, -2* plants were collected under moderate light conditions (120 μmol of photons m^–2^ s^–1^) (ML) and after 3 h of high light (500 μmol of photons m^–2^ s^–1^) (HL). **(A)** Normalized area (Area/FV) above the chlorophyll fluorescence induction curve measured after 15 min of dark incubation. This value estimates the number of available electron carriers per PSII reaction center. **(B)** Quantum electron transport yield from QA to PQ pool (ΦET2o), and **(C)** until the PSI electron acceptors (ΦRE1o). These measures were performed with Handy-PEA (Hansatech Instruments). Whiskers and box plot shows the minimum, first quartile, median, average, third quartile, and maximum of each data set (*n* = 12 biologically independent samples). Superscript letters are used to indicate statistically different groups by paired Student’s *t*-test (*p* < 0.05).

The ETC capacity, before and after the high light treatment, was also estimated using fast chlorophyll *a* fluorescence (JIP-test) by normalizing the area above curve over variable fluorescence [Area/(F_M_-F_O_)] (Strasser et al., 2010; Kalaji et al., 2014a, b). This area positively correlates with the number of turnovers of the QA site of PSII before being fully closed. Since each turnover corresponds to a single electron injected in the ETC, the area offers a proxy of the number of available electron acceptors per PSII. These acceptors are internal to PSII, pheophytin and QA, or external, PQ molecules of the photoactive plastoquinone pool, the cyt *b6f* complex and plastocyanin. Under moderate light, the electron capacity estimated by the normalized area, was bigger in *abc1k3* than in WT (17 ± 2), while it was smaller in both *abc1k1* lines (15 ± 1; 14 ± 1). In the two *abc1k1/abc1k3* lines the estimated electron capacity (18 ± 2; 20 ± 3) was comparable to WT (Figure 4A). A second measurement was performed on leaves sampled after 3 h of high light, in this sample, compared to the moderate light condition, the availability of the electron acceptors per PSII remained essentially unchanged in WT (19 ± 2) and in both *abc1k1/abc1k3* lines (19 ± 2; 18 ± 3). In the two *abc1k1* lines, after high light treatment, the electron transport capacity was diminished (13 ± 1; 11 ± 2) compared to the moderate light conditions. In contrast, both *abc1k3* lines showed a tendency toward an increase in electron carriers (22 ± 2; 24 ± 3) when comparing leaves sampled after 3 h of high light with those harvested in moderate light condition (Figure 4A).

To obtain an indication of the impact of the mutations on different components of the electron transport chain, we calculated the quantum yields of the electron transport flux from QA^–^ to PQ (ΦET2o) and to the PSI electron acceptors (ΦRE1o) by analyzing chlorophyll fluorescence inductions of JIP-curves at 3 ms (FJ) and 30 ms (FI).

In leaves collected under moderate light, ΦET2o was lower in *abc1k1/abc1k3* and *abc1k1* compared to WT and *abc1k3*. After challenging the plants with 3 h of high light, the measurement revealed that ΦET2o dropped in all lines, with the most severe decrease in *abc1k1* lines followed by *abc1k1/abc1k3* (Figure 4B).

The quantum yield of electron transport to PSI final acceptors (ΦRE1o), in plants maintained under moderate light, was similar in WT and *abc1k1/abc1k3*, lower in *abc1k1* and higher in *abc1k3*. After 3 h of high light, ΦRE1o in *abc1k1*diminished even further, while it remained essentially unchanged in *abc1k1/abc1k3* when compared to moderate light. Therefore, *abc1k1/abc1k3* quantum yield was comparable to the WT in both light condition. For this parameter, the double mutation appears to partially attenuate the photosynthetic defects due to the *abc1k1* mutation. Finally, after high light, ΦRE1o in *abc1k3* did not change compared to moderate light, thus being always higher than in the WT (Figure 4C).

To verify whether the recovery of photosynthetic parameters in *abc1k1/abc1k3* as well as the higher photosynthetic capacities measured in *abc1k3*, were due to an increased cytochrome *b6f* activity, the turnover rate was measured (Finazzi et al., 2002). The measurement showed that there was no negative impact on the level of activity among all the tested lines (Supplementary Figure 2).

Together, these results indicate that the mutation of *abc1k3* has no negative impact the electron transport between the photosynthetic complexes, since the transport efficiency in this mutant line was elevated also after exposure to high light. Conversely, the electron transport in *abc1k1* was impaired in the moderate light condition and worsened upon exposure of the plants to 3 h of high light. The double mutation of *abc1k1* and *abc1k3* resulted in a partial recovery of the electron transport and NPQ to WT levels when compared to *abc1k1.*

### Mutation of abc1k3 Has No Effect on the Photoactive PQ Pool Size

The level of NPQ and the electron acceptor capacity of the ETC may be related to the size of the photoactive PQ pool (Block et al., 2013; Pralon et al., 2019). Therefore, we examined whether the partial rescue of the electron transport and NPQ capacities in *abc1k1/abc1k3* compared to *abc1k1* can be attributed to the size of the photoactive plastoquinone pool (i.e., the number of PQ molecules readily available per PSII). For this, we measured the total PQ (nmol cm^–2^) and the relative photoactive PQ pool (in% of total PQ pool) in 4–5 weeks old plants under moderate light and after 3 h of high light exposure. The photoactive PQ pool is defined as the fraction of total PQ that is rapidly reduced during a saturating light pulse and oxidized when the sample is exposed to far-red light. To measure this fraction, PQH_2_ and PQ amounts were analyzed by HPLC-MS on leaves illuminated either with a strong light flash in order to completely reduce the photoactive PQ pool, or with far-red to obtain its complete oxidation (Kruk and Karpinski, 2006; Block et al., 2013; Ksas et al., 2018; Pralon et al., 2019). The difference between the amount of reduced PQ after the saturating flash and that measured after far-red illumination determines the photoactive PQ pool.

**FIGURE 5:**
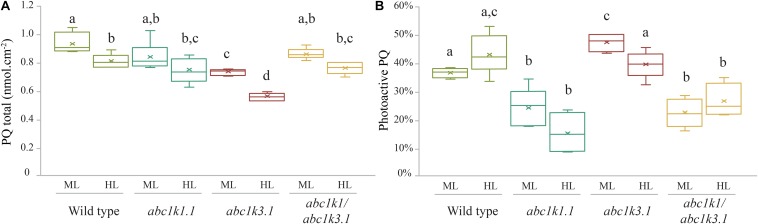
Photoactive PQ pool is smaller in all the lines containing the *abc1k1* mutation. **(A)** Total plastoquinone content (nmol cm^–2^) and **(B)** photoactive plastoquinone pool (as% of total PQ) were analyzed from leaves of Wild type, *abc1k1.1*, *abc1k3.2*, and *abc1k1/abc1k3.2* under moderate light and after 3 h high light. The photoactive plastoquinone pool was determined by the difference of the plastoquinol (PQH_2_) amount measured when the PQ pool is maximally reduced by strong white light pulse (15 s at 2,000 m^–2^ s^–1^), and the amount of PQH_2_ measured when the PQ pool is fully oxidized after far-red illumination (2 min at 5.5 μmol of photons m^–2^ s^–1^). Whiskers and box plot shows the minimum, first quartile, median, average, third quartile, and maximum of each data set (*n* = 4 biologically independent samples). Superscript letters are used to indicate statistically different groups by paired Student’s *t*-test (*p* < 0.05).

Total plastoquinone levels (photoactive PQ pool + non-photoactive PQ pool) in *abc1k1* and in *abc1k1/abc1k3* lines were similar when compared to WT and decreased only slightly after 3 h of high light exposure. Conversely, the total PQ level was lower in *abc1k3* compared to WT (Figure 5A). The photoactive PQ pool (photoactive PQ/total PQ) measured in *abc1k3* was larger under moderate light condition and identical to the WT after 3 h of high light (Figure 5B). Whereas, in *abc1k1* after 3 h of high light, the photoactive PQ pool was strongly diminished compared to WT. Although the double mutant was not severely impaired in either ETC capacity or NPQ (Figures 2, 4), the relative photoactive PQ pool, measured in plants grown under moderate light, in *abc1k1/abc1k3* was unexpectedly small and similar to the one of *abc1k1*. High light had no negative effect on the size of the photoactive PQ pool in the *abc1k1/abc1k3* double mutant. However, after 3 h of high light, its photoactive PQ pool size was smaller than that of the WT or *abc1k3*, and comparable to *abc1k1* (Figure 5B).

The analysis of the photoactive PQ pool suggests that the complementation of the *pgr6*(*abc1k1*) photosynthetic phenotype, induced by the *abc1k3* mutation, does not simply arise from a change of the amount of PQ readily available at PSII.

### Mutation of ABC1K1 and ABC1K3 Impacts the Kinetics of PQ Re-oxidation in the Dark

**FIGURE 6:**
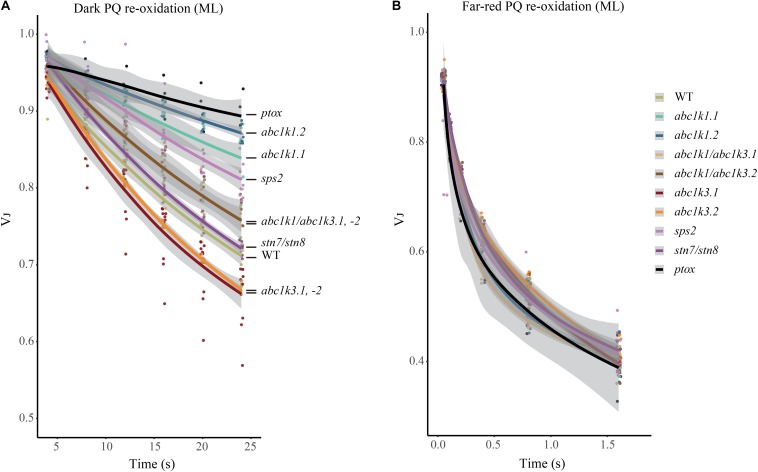
Mutations of ABC1K1 and ABC1K3 influence the dark re-oxidation kinetic of the photoactive plastoquinone. The relative fluorescence after 3 ms (VJ) is plotted over the time from the previous saturating pulse. The sample was incubated in dark **(A)** or with far-red light **(B)**. Kinetics of the re-oxidation are shown by interpolation of the data points with a logarithmic curve with the deviation of the model in gray (R-Studio) for wild type (WT) *abc1k1.1, -2, abc1k3.1, -2*, and *abc1k1/abc1k3.1, -2, sps2, stn7/stn8, ptox* plants grown under moderate light (ML). These measures were performed with Handy-PEA (Hansatech Instruments) on detached leaves incubated for 10 min in dark.

The redox state of PQ in the light is dependent on the activity of the PSII, which will reduce the photoactive pool, and on that of the cytochrome *b6f*, which oxidizes the PQ pool transferring electrons along the ETC toward PSI. However, the photosynthetic complexes are inactive in the dark and therefore the redox state of PQ is mostly dependent on light-independent electron routes alternative to the cytochrome *b6f*. In the transition from light to dark the photoactive PQ pool will tend to start in a reduced form and be re-oxidized. The principal actor of this re-oxidation is PTOX. This enzyme, in Arabidopsis, is mostly located in the stroma lamellae fraction of the thylakoid membrane (Joet et al., 2002; Lennon et al., 2003; Houyoux et al., 2011). PSII being more abundant in the grana stacks, and considering the timescale of the mobility of PQ (Kirchhoff, 2014), we may assume that a large portion of the photoactive PQ pool is located within the grana stacks. Therefore, in order to be oxidized by PTOX the photoactive PQ has to migrate from the grana stacks to the stroma lamellae and then return to the grana stacks. Considering this, the kinetics of the oxidation of the photoactive PQ pool represents a proxy of the mobility of the PQ across the different portions of the thylakoid membrane. To estimate the redox state of the photoactive PQ pool we used the rapid chlorophyll *a* fluorescence induction, we based the analysis on the relative fluorescence at 3 ms (VJ). It has been shown that the fluorescence recorded at this time interval correlates with the redox state of the photoactive PQ pool (Strasser et al., 2010; Kalaji et al., 2014a). A high VJ value is recorded in samples where the photoactive PQ pool is mostly reduced, and it decreases with its oxidation (Tóth et al., 2007). The chlorophyll *a* fluorescence induction was measured at increasing time intervals of dark incubation after a saturating light pulse. The results show that the measured oxidation, which appears to be almost completely PTOX dependent, is faster in the *abc1k3* mutants, while severely impaired in *abc1k1* (Figure 6A).

A limitation of total PQ, as in the *sps2* mutant (Block et al., 2013; Pralon et al., 2019), resulted in a slower oxidation in the dark as well. The PQ oxidation in the *abc1k1/abc1k3* double mutant progressed more rapidly than in the *abc1k1* single mutant (Figure 6A). The dark re-oxidation of the photoactive PQ pool is independent on the photosynthetic ETC. In fact, all the tested mutant lines displayed the same kinetics when the PQ oxidation was performed by the cytochrome *b6f*, as observed when PSI was excited with far-red light (Figure 6B). The kinetics of PQ oxidation in the dark may be influenced by the level of accumulation of the PTOX protein (Ivanov et al., 2012). We therefore used immunodetection to test the PTOX accumulation in total protein samples from the different mutants. The result showed no differences at the protein level, which appeared to be uniform among the lines (Supplementary Figure 3). After 3 h under high light the dark re-oxidation of the photoactive PQ in the *abc1k1* mutant is almost completely blocked, and it is slower overall in all lines analyzed. Once again, the defect was milder in the *abc1k1/abc1k3* double mutant (Supplementary Figure 4). It is worth noting that after 3 h of high light the oxidation kinetics under far-red light were also affected in *abc1k1*, suggesting that 3 h of high light exposure induce a perturbation of the ETC, consistently with the previous report (Pralon et al., 2019).

This experiment shows that *abc1k1* is impaired in the regulation of the photoactive PQ redox state independently of the activity of the ETC. Considering the specific localization and identical protein levels of PTOX, this supports a model of limited mobility of PQ. Interestingly, said defect is partially complemented by *abc1k3* mutation.

### Major Thylakoid Membrane Protein Phosphorylation and State Transitions Are Partially Restored in abc1k1/abc1k3

A smaller photoactive PQ pool (Figure 5B) should be prone to over-reduction or at least to “mimic” a condition of over-reduction as fewer PQ molecules are available (Figure 4B). This would be expected to perturb state transitions (Bellafiore et al., 2005; Shapiguzov et al., 2010, 2016; Tikkanen et al., 2012; Trotta et al., 2016; Pralon et al., 2019). Cytochrome *b6f* activity is dependent on the redox state of PQ and regulates the activation of STN7, the principal kinase involved in LHCII phosphorylation. Therefore, we evaluated the phosphorylation status of the major thylakoid membrane proteins by immunodetection. Moreover, we measured the re-allocation of the mobile light harvesting complex II (LHCII) between the two photosystems by room temperature chlorophyll fluorescence in *abc1k1/abc1k3* during state 1 to state 2 transition induced by changes in the light spectrum. Both approaches were used as proxies to assess the redox state of the PQ pool *in vivo*.

**FIGURE 7:**
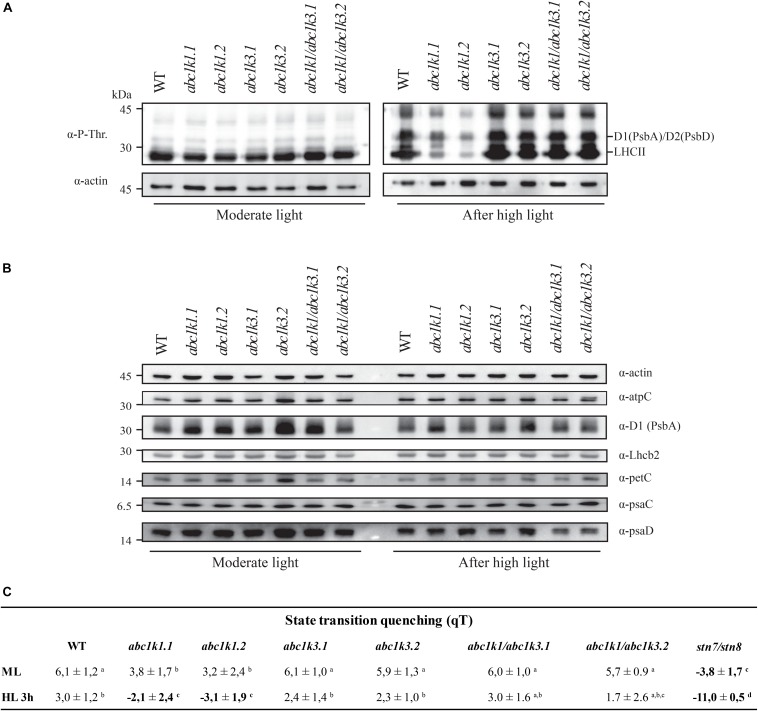
Double mutant maintains thylakoid protein phosphorylation and state transitions after high light. **(A)** Total protein extracts of wild type (WT), *abc1k1.1, -2, abc1k3.1, -2*, and *abc1k1/abc1k3.1, -2* light-exposed leaves were separated by SDS PAGE, transferred on nitrocellulose membrane and decorated with anti-phosphothreonine antibody. The main thylakoid phospho-proteins are indicated on the right according to their size. Core photosystem II proteins D1 (PsbA) and D2 (PsbD) are indicated together due their poor resolution. **(B)** The accumulation of the principal photosynthetic complexes was assessed using antibodies against specific subunits of each complex: anti-Lhcb2 for the major LHCII, anti-D1 (PsbA) for PSII, anti-PetC for cytochrome *b6f*, anti-PsaD and anti-PsaC for PSI, and anti-AtpC for ATP synthase. Actin signal is shown as a loading control. **(C)** Fluorescence quenching related to the state transitions (qT) of wild type (WT), *abc1k1.1, -2, abc1k3.1, -2*, and *abc1k1/abc1k3.1, -2* under moderate light (120 μmol of photons m^–2^ s^–1^) (ML) and after 3 h of high light (500 μmol of photons m^–2^ s^–1^) (HL). qT was calculated from the maximal chlorophyll fluorescence measured after 10 min exposure to red light (660 nm) supplemented with far-red illumination (720 nm) “State 1” (FMST1) or to pure red light “State 2” (FMST2). Quenching related to state transition was calculated as qT = (FMST1 – FMST2)/FM. Each value represents the average of a pot containing 2–3 plants. Superscript letters are used to indicate statistically different groups (*p* < 0.05) by paired Student’s *t*-test.

The phosphorylation status of the thylakoid protein was assessed by anti-phosphothreonine immunoblotting on total protein extracts from leaves collected under moderate light and after 3 h high light exposure. Under moderate light, the thylakoid phosphoprotein pattern was similar among all the lines tested (Figure 7A). Compared to the moderate light, after 3 h of high light exposure PSII core proteins D1 (PsbA) and D2 (PsbD) were only slightly more phosphorylated in *abc1k1*, while their phosphorylation increased markedly in WT, *abc1k3* as well as in *abc1k1/abc1k3*. Similarly, after the high light stress, the LHCII proteins were highly phosphorylated in *abc1k3, abc1k1/abc1k3*, and WT, while the LHCII phosphorylation was clearly lower in *abc1k1* (Figure 7A). This suggests that, despite the shortage of photoactive PQ in *abc1k1/abc1k3* (Figure 5B), the STN7 kinase maintains its activity toward LHCII even after exposure to high light. Furthermore, neither the change in total PQ nor in the photoactive fraction had an impact on the abundance of selected photosynthetic proteins after 3 h of high light (Figure 7B).

To determine whether thylakoid protein phosphorylation observed in *abc1k1/abc1k3* correlated with the ability to perform state transitions we followed and measured room temperature chlorophyll *a* fluorescence kinetics on dark-adapted plants by switching red light supplemented with far-red light (State 1) to red light only (State 2). The quenching (fluorescence decline) caused by state transitions (qT), was calculated as the difference between the maximal fluorescence (FM) after “State 1” illumination (FM_ST1) and the one after “State 2” light (FM_ST2) normalized on maximal fluorescence (FM) (qT = (FM_ST1 – FM_ST2)/FM), which reflects the dissociation of antenna from PSII and its association with PSI.

Under moderate light, the qT in all genotypes was comparable to the WT, indicating their ability to perform transition from State 1 to State 2 and only *abc1k1* lines appeared to be slightly impaired (Figure 7C). *stn7/stn8*, which is completely unable to perform state transitions, was used as a negative control. After 3 h of high light exposure, *abc1k3* and WT maintained their capacity to perform state transitions, while *abc1k1* lines were defective in state transitions. After 3 h of high light, state transitions in *abc1k1/abc1k3* lines exhibited a level of quenching comparable to WT and *abc1k3* (Figure 7C). This shows that LHCII phosphorylation, and thus the activity of the STN7 kinase, which is maintained in *abc1k1/abc1k3* mutants, allows the state transitions after high light exposure.

## Discussion

The photosynthetic apparatus has to adapt to photo-oxidative stress induced by excess light in order to prevent thylakoid membrane damage and maintain photosynthetic efficiency. Photo-protective strategies comprise adjustment of electron transport capacity (ETC) (Rochaix, 2011), equilibration of energy between photosystems (state transitions) (Rochaix, 2007) and induction of NPQ (Müller et al., 2001; Ruban, 2016). All three mechanisms are directly or indirectly related to the activity of the plastoquinone as an electron carrier. Recently, it has been demonstrated that ABC1K1 is implicated in photosynthesis regulation by homeostasis of photoactive PQ under high light (Pralon et al., 2019). In this study, we describe the involvement of ABC1K3 (Lundquist et al., 2013; Martinis et al., 2013; Huang et al., 2015), a close homolog of ABC1K1, in the same process.

We tested two *abc1k3* mutant lines for their ability to induce NPQ under increasing light intensities. Contrary to *abc1k1*, this mutation did not cause any perturbation in NPQ induction when compared with the WT (Figure 2). A previous report shows that, during several days of exposure to high light (500 μmol of photons m^–2^ s^–1^), *abc1k3* had a tendency to induce slightly less NPQ compared to WT, however this difference was not statistically significant nor constant over the time course (Martinis et al., 2013). This suggests that the NPQ parameter alone is not sufficient to discriminate the photosynthetic adaptation of *abc1k3* from WT. In fact, the differences between WT and *abc1k3* are significant only when the photosynthetic ETC capacity is analyzed in detail (Figures 3, 4). To further address the role of *abc1k3* in the photosynthetic regulation, we crossed this mutant with *abc1k1* to obtain *abc1k1/abc1k3* double mutant plants (Figure 1). The *abc1k3* mutation was capable to partially alleviate the NPQ defect observed in *abc1k1*; while the NPQ level in *abc1k1/abc1k3* was higher than in the single *abc1k1* mutant, it remained lower than in the WT (Figure 2).

In order to confirm that the NPQ perturbation is due to a lack of transport through the ETC, we measured the number of electrons transported per PSI after a saturating light pulse by analyzing the lag time of PSI oxidation. As expected from previous reports (Shikanai et al., 1999; Martinis et al., 2014; Pralon et al., 2019), *abc1k1* transfers less electrons to PSI, and the difference compared to the WT increases after exposure to 3 h high light. On the contrary, *abc1k3* was not impaired and appeared capable of maintaining a high level of electron transfer after 3 h of high light (Figure 3). In *abc1k1/abc1k3*, the electron transport capacity was still lower than the WT, however, more electrons were transferred per PSI compared to *abc1k1* and the decrease after 3 h of high light was comparable to the one observed in the WT (Figure 3). This finding suggests that the defect in the photosynthetic electron carriers of *abc1k1* was partially rescued in *abc1k1/abc1k3*, presumably by increasing the efficiency of the electron transfer in the ETC. In a previous report, the limitation of the electron transport capacity observed in *abc1k1* was linked to a depletion of the photoactive PQ pool (Pralon et al., 2019). However, the decrease in the number of electrons transported to PSI appeared to be more severe than the measured decrease in the size of the photoactive PQ pool. Therefore, it was hypothesized that the PQ mobility in the ETC plays an additional role in limiting the electron transport capacity. To investigate this hypothesis the energy fluxes along the ETC were analyzed by rapid fluorescence induction curves. The estimation of the number of electrons present in the ETC before saturation, expressed as the Area/FV (Figure 4A), was consistent with the P_700_ oxidation analysis (Figure 3). In fact, already under moderate light the normalized area was smaller in *abc1k1* mutant and larger in *abc1k3* in comparison to WT, consistent with *abc1k1* having limited electron transport and *abc1k3* having more carriers than the WT. In this condition, the *abc1k1/abc1k3* double mutant had an Area/FV value in between those of the two single mutants, suggesting once again, a partial recovery of the photosynthetic electron transport. Upon exposure to high light only the *abc1k1* mutant showed a decrease in the number of available carriers; while all the other lines had a tendency to increase the Area/Fv value, compared to moderate light, indicating a better, or at least unchanged, electron transport capacity (Figure 4A). By analyzing the induction curve’s principal steps, we can obtain hints regarding the different components that may be affected in the ETC. The first step, at 3 ms from the start of the saturating flash, was reported to be dependent of the QB redox state at the PSII and therefore linked to the redox state of the photoactive PQ pool (Tóth et al., 2007), which is also affected by the size of the photoactive PQ pool (Pralon et al., 2019). From the fluorescence value at 3 ms (FJ) it is possible to calculate the ΦET2o, the efficiency of the electron transport between QA and QB (which depends on the status of the photoactive PQ pool) (Strasser et al., 2010; Kalaji et al., 2014a, b). We observed that the ΦET2o is lower both in *abc1k1* and in *abc1k1/abc1k3*, suggesting a similar defect in the photoactive PQ pool. However, the defect appears to be somewhat milder in the double mutant compared to *abc1k1* (Figure 4B). The second step in the fluorescence rise occurs at 30 ms (FI), and the level of the fluorescence recorded at this step is linked to the electron transport to the PSI final acceptors in the OJIP model (Strasser et al., 2010; Kalaji et al., 2014a, b). From the FI value is also possible to calculate the electron transport efficiency, defined as ΦRE1o. Comparison of this efficiency revealed that *abc1k1* has a lower efficiency compared to the WT but that was not the case for *abc1k1/abc1k3*. In the latter, the ΦRE1o was the same as in the WT in moderate light, and was less affected upon the exposure to 3 h high light compared to *abc1k1* (Figure 4C). The *abc1k3* single mutation did not create any measurable defect in electron transport. On the contrary, the efficiency of the transport to PSI acceptors appeared to be even higher than in the WT, both under moderate light and after exposure to high light (Figure 4C). This suggests that the mutation of the *ABC1K3* gene leads to an increased efficiency in the electron mobility between PSII and PSI.

To support the fluorescence induction results we biochemically measured the size of the photoactive pool in the mutants exposed to moderate light and to high light. Consistent with the biophysical observations, the photoactive PQ pool was smaller in both *abc1k1* and *abc1k1/abc1k3* compared to the WT in moderate light condition (Figure 5B). Exposure to 3 h of high light had a limited effect on the size of the photoactive PQ pool in the four tested lines (Figure 5B). However, while the WT and the double *abc1k1/abc1k3* mutant display a tendency toward the increase of the photoactive PQ after 3 h of high light, we detected a slight decrease of the photoactive PQ pool in *abc1k1* as previously reported (Pralon et al., 2019). The depletion observed in this report was not significant as it was in the previous report, this may suggest that the photoactive PQ pool homeostasis relies on multiple factors (e.g., thylakoid organization, lipid distribution) and that ABC1K1, despite its prominent role, is not the only factor regulating the photoactive PQ pool size. A similar depletion of the photoactive PQ pool after 3 h of high light, was observed in *abc1k3* even though the relative photoactive PQ pool size was still larger than in *abc1k1*, and comparable to the WT (Figure 5B). On the contrary, the *abc1k1/abc1k3* double mutant constitutively displayed a small photoactive PQ pool that was not depleted by the exposure to high light (Figure 5B). This leads to the conclusion that the photoactive PQ pool size *per se* has a limited influence on photosynthetic efficiency, and that the photosynthetic defect becomes symptomatic only when PQ limitation is associated with an additional impairment in its mobility. Impaired PQ mobility may be the cause affecting the reoxidation of the photoactive PQ by PTOX, since it may require an exchange between grana stacks and stroma lamellae (Figure 6A; Stepien and Johnson, 2018). Furthermore, it would affect the exchange between the photoactive pool and the reservoir stored in the PGs necessary to maintain the photoactive PQ pool size (Pralon et al., 2019). We cannot exclude that the defect in the mobility and exchange between the different PQ pools stems from a defect in the thylakoid membrane composition. In fact, it has been previously reported that the mutation of *abc1k1* results in a lower amount of several prenyl lipids (Martinis et al., 2014) compared to the WT under control growth conditions (150 μmol of photons m^–2^ s^–1^) as well as after several days of growth in high light (500 μmol of photons m^–2^ s^–1^). However, the *abc1k3* mutant has a similar defect in the level of accumulation of these lipophilic compounds (Martinis et al., 2013). Therefore, no obvious correlation between the photosynthetic defect and the amount of the principal chloroplast lipids can be drawn at this stage.

**FIGURE 8:**
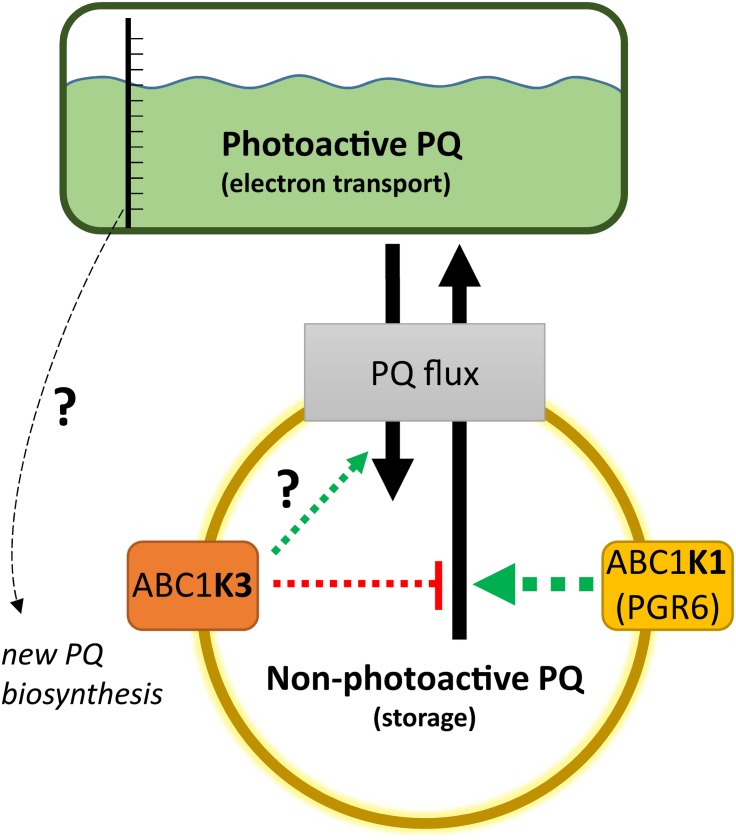
Working model on the activity of ABC1K1 and ABC1K3 on the plastoquinone (PQ) redistribution. The two atypical kinases are enriched in the plastoglobule proteome, therefore presumably in the proximity of the non-photoactive PQ pool. The activity of the ABC1K1 promotes the mobilization of PQ toward the photoactive pool, while ABC1K3 counteracts this mobilization thus favoring the accumulation of PQ in the non-photoactive pool. In the absence of both kinases the PQ tends to accumulate in the non-photoactive pool, therefore a positive regulation of ABC1K3 on the flux from the photoactive to the non-photoactive pool is not essential, as highlighted by the question mark, but cannot be excluded in this model. The observation that in absence of both kinases PQ tends to accumulate in the non-photoactive pool suggests a prominent role of ABC1K1 in the PQ flux regulation (larger arrow). A not yet identified sensor of the size and/or the redox state of the photoactive PQ pool may serve to regulate the level of PQ biosynthesis. When the relative size of the photoactive pool is larger, and the electron transport more efficient as in the *abc1k3* mutant, the signal promoting PQ biosynthesis may be dimmed resulting in a lower amount of total PQ. The dashed arrows represent that the regulation of the PQ fluxes, mediated by the two atypical kinases, is likely to be indirect. Although the effect on PQ flux is opposite, the presented working model cannot exclude that the two atypical kinases act via separate mechanisms and that the impact observed on PQ mobility is a consequence of the alteration of their primary targets.

A lower amount of total PQ, which decreased after 3 h of high light, was observed in *abc1k3* (Figure 5A). This decrease may be an indirect effect of the increased photosynthetic electron transport observed in *abc1k3* compared to WT. A possible explanation is that the amount or the redox state of PQ in the photoactive pool act as a signal to regulate PQ biosynthesis. This is supported also by previous reports showing a correlation between PQ biosynthesis and increase in light intensity (Zbierzak et al., 2010; Ksas et al., 2015). Consistent with this hypothesis, the increased relative PQ accumulation in the photoactive pool, observed in the *abc1k3* mutant under moderate light (Figure 5B), would alter this signal and therefore limit the biosynthesis resulting in a lower amount of total PQ (Figures 5A, 8).

A crosstalk between photoactive PQ pool and biosynthesis would ensure the coupling of the photosynthetic electron transport efficiency with the production of a PQ reserve in the non-photoactive pool allowing fast adaptation to changing environmental conditions. It is necessary to point out that the decrease of total PQ after 3 h of high light was likely to be transitory since, under permissive light intensity, *abc1k3* was capable of restoring the total PQ amount to WT levels as previously shown (Martinis et al., 2013). We cannot exclude that ABC1K1 and ABC1K3 play an active role in this signaling pathway, through still unidentified targets, therefore the mutation of these two kinases would result in a combined effect on the source of the signal, the photoactive PQ pool, as well as on the transmission of said signal.

Another feature linked to the *abc1k1* mutation was the loss of thylakoid protein phosphorylation. These phosphorylation events are mostly dependent on STN7 and STN8 kinases, activities of which depend on the redox status of the PQ pool (Aro and Ohad, 2003; Pesaresi et al., 2010; Puthiyaveetil, 2011; Puthiyaveetil et al., 2012). The mutation of *abc1k3* in the *abc1k1* background was sufficient to re-establish the phosphorylation of the thylakoid proteins to WT level after 3 h of high light. This observation has an important implication to understand the factors regulating the redox state of the photoactive PQ pool. In fact, the PQ pool redox state appears to be dependent also on the exchanges between the photoactive pool and storage sites and not only on the size of the photoactive pool and the activity of the ETC. We cannot exclude that protein phosphorylation has a positive feedback loop effect; in fact, the phosphorylation of the thylakoid proteins may favor their mobility in the membrane and by doing so increase also the mobility of the PQ between different portions of the thylakoid membrane (Kirchhoff et al., 2000; Fristedt et al., 2009; Kirchhoff, 2014). We also cannot exclude the involvement of the phosphorylation of other, less evident, targets such as CURT1b, the phosphorylation of which is also dependent on STN8 kinase (Trotta et al., 2019). By their phosphorylation level, the proteins imbedded in the thylakoid membrane may change the overall conformation of the membrane system and by this also the mobility and exchange of the PQ, and other lipids, between the photoactive pool and the reservoir (Armbruster et al., 2013). Moreover, ABC1K1 and ABC1K3 are also predicted to function as kinases; therefore, they may phosphorylate yet unknown target proteins leading to the regulation of the photosynthetic activity and potentially influencing membrane fluidity and/or thylakoid protein organization (Yokoyama et al., 2016).

The results lead us to propose the following model for the action of ABC1K1 and ABC1K3, being two kinases located at the plastoglobule (Vidi et al., 2006; Lundquist et al., 2012; Martinis et al., 2013, 2014). The role of ABC1K1 would be to promote the release, or the exchange of the PQ between the storage and the photoactive pool, while ABC1K3 would act in limiting such diffusion blocking this PQ flux (Figure 8). This model would explain the slightly bigger size of the photoactive pool in *abc1k3* and also the difference between *abc1k1* and *abc1k1/abc1k3*, the latter having a better photosynthetic performance since it is missing the ABC1K3 protein that would otherwise act as a “brake” to the PQ supply to the photoactive pool (Figure 8). To fully explain the observed results, we also have to assume that, without the activity of ABC1K1, the PQ tends to over-accumulate in the non-photoactive pool thus leading to a depletion of the photoactive pool. In this regard, the role of ABC1K1 is crucial to maintaining the photosynthetic efficiency by promoting the movement and accumulation of the PQ against its “passive” distribution. In contrast ABC1K3, acting as a brake to the mobilization of the non-photoactive pool, may have a more important role in other processes and phases of plant development when lipids need to be efficiently accumulated in the reserve compartments (e.g., senescence, fruit maturation). Previous reports describe a direct interaction between ABC1K1 and ABC1K3 (Lundquist et al., 2013). It is possible to hypothesize that this interaction allows a reciprocal regulation of the two kinases. For instance, a negative regulation of ABC1K3, mediated by ABC1K1, could partially explain the results of this report. In this scenario, the *abc1k1* mutation would cause a deregulation, or over activation, of the ABC1K3 kinase and this would then cause the photosynthetic defect. Following this model, the removal of ABC1K3, as in the double *abc1k1/abc1k3* mutant, would partially rescue the defect caused by the first mutation. It has to be underlined that the physiological state of the double mutant *abc1k1/abc1k3* is still not optimal and that the absence of ABC1K3 only partially rescues the photosynthetic phenotype. This observation is thus consistent with previous reports showing that the double mutant, lacking both kinases, displays a stunted phenotype under prolonged stress conditions to which is unable to adapt (Lundquist et al., 2013).

## Conclusion

In conclusion, we show that the *abc1k3* mutation allows a partial recovery of the *pgr6/abc1k1* phenotype. Consequently, ABC1K1 and ABC1K3 act in an opposite manner in order to cope with short-term high light. Therefore, we suggest that the system of the atypical kinases ABC1K1 and ABC1K3 allows a dynamic regulation of the PQ pool mobility and availability, which is fundamental for the plant to cope with variations in environmental conditions. In the working model ABC1K3 would act by limiting the distribution of PQ to the photoactive pool, while ABC1K1 acts in the opposite way. The role of ABC1K1 would be prominent considering that, without any regulation, the relative amount of PQ in the photoactive pool is lower, as is the case for the double *abc1k1/abc1k3* mutant. The opposing activities of these two atypical kinases would allow to equilibrate the amount of PQ, and potentially other lipids, between the storage compartments and the thylakoid membrane. Finally, a hypothetical signaling mechanism from the photoactive PQ pool to the PQ biosynthesis could explain the decreased amount of total PQ observed in *abc1k3* (Figure 8). Overall, the ABC1K1/ABC1K3 mechanism, by regulating lipid partitioning, could be important to allow plastid plasticity that is essential for the progression into different developmental stages of the plant life.

## Data Availability Statement

The raw data supporting the conclusions of this article will be made available by the authors, without undue reservation, to any qualified researcher.

## Author Contributions

TP, VS, PL, and FK designed the experiments. TP, JC, RP, BK, VS, PL, MH, and GF performed all the experiments. TP, VS, PL, MH, GF, and FK contributed to the analysis and the interpretation of the results. TP, PL, MH, GF, and FK wrote the manuscript.

## Conflict of Interest

The authors declare that the research was conducted in the absence of any commercial or financial relationships that could be construed as a potential conflict of interest.

## Footnotes

1 http://arabidopsis.info

2 http://www.psi.cz
