# Supplementary material for: Mutation of the Atypical Kinase ABC1K3 Partially Rescues the PROTON GRADIENT REGULATION 6 Phenotype in Arabidopsis thaliana
Source: Front Plant Sci. 2020 Mar 25;11:337. doi: 10.3389/fpls.2020.00337 (PMC7109304; doi:10.3389/fpls.2020.00337)
Supplement: Supplementary file 1 [file Data_Sheet_1.PDF]

```
               10        20        30        40        50        60
ABC1K1 MESIHCNSLLNPNFSLNQRRRRINHAVLNRRDALLRSLNAVELRRSRTFSAVRTSNFSVT
       :  .  .::   ...:               :.:    . ... .:. ::. :
ABC1K3 MSLVVGQSL---GLTLVG-------------DGLSLRNSKINVGKSKFFSVNRRR----L
                  10                     20        30            40

               70        80        90       100          110
ABC1K1 AAATDVGGRNSTDASVMTTAMSGVERGVRVGKSSSALEQLDIER---GVCVPFRKYSPET
       : :. : .: . :... . . :.   .:   . ..  ..:. :    :  .    ::::
ABC1K3 ARAALVQARPKEDGAAASPSPSSRPASVVQYRRADLADDLQAEARALGRAIDASIYSPEL
               50        60        70        80        90       100

       120       130       140       150       160       170
ABC1K1 VRSKVLESRGAVVSLVSRGVEIVWTLGLYWSTLTYDFLVGRDEEVVPFRARQLRNLLCNL
       .  :     .   . . :..::. .:: .   :  :   :  :. .  :: .:: ..  :
ABC1K3 IARK---HGSQPFKALRRSLEILGALGGFALKLGIDQKQGNLEKNMKKRAIELRRIFTRL
                 110       120       130       140       150

       180       190       200       210       220       230
ABC1K1 GPSFIKAGQVLANRPDIIREDYMNELCILQDDVPPFPNEVAFNIIEEELGQPLENIFSKI
       ::.:.: :: :..:::.   ::..::  ::: .: ::.  ::  ::.::   ::.:::..
ABC1K3 GPTFVKLGQGLSTRPDLCPPDYLEELAELQDALPTFPDAEAFACIERELDLSLETIFSSV
       160       170       180       190       200       210

       240       250       260       270       280       290
ABC1K1 SSQTIAAASLGQVYRATLRATGEDVAIKVQRPQIEPIIYRDLFLFRTLASFLNGFSLQKL
       : . ::::::::::.: :: .:. ::.::::: ::  :  :..:.: .....: . .. .
ABC1K3 SPEPIAAASLGQVYKAQLRYSGQVVAVKVQRPGIEEAIGLDFYLIRGVGKLINKY-VDFI
       220       230       240       250       260       270

       300       310       320       330       340       350
ABC1K1 GCNAELIVDEFGEKLLEELDYTLEARNIEDFLENFKDDPTVKIPGVYKNLCGPRVLVMEW
         ..  ..:::. .. .::.:. ::.: . : . . :   : .: .. .  . .::.:::
ABC1K3 TTDVLTLIDEFACRVYQELNYVQEAQNARRFKKLYADKADVLVPDIFWDYTSRKVLTMEW
        280       290       300       310       320       330

       360       370       380       390       400       410
ABC1K1 IDGIRCTDPQAIKDAGIDLNGFLTVGVSAALRQLLEFGLFHGDPHPGNIFAMQDGRIAYV
       ..: .  ..  ::.. :. .  ....:.. .::::::.:.::.::::::..:  ::..:..
ABC1K3 VEGTKLNEQLAIESQGLKVLDLVNTGIQCSLRQLLEYGFFHADPHPGNLLATPDGKLAFL
        340       350       360       370       380       390

       420       430       440       450       460       470
ABC1K1 DFGNVAVLSQQNKQILIDAVVHAVNEDYGEMANDFTRLGFLAKDTDVSPIVPALEAIWQN
       ::: ..   .. .  .:  ::: ::.::  :: :.  : ::. :.::.::.:::. ....
ABC1K3 DFGMMSETPEEARFAIIGHVVHLVNRDYEAMARDYYALKFLSPDVDVTPIIPALRDFFDD
        400       410       420       430       440       450

       480       490       500       510       520       530
ABC1K1 SAGKGLADFNFRSVTGQFNKLVYDFPIRIPERFSLVIRSLLTQEGICFTLKPDFKFLEVA
       . .  ....::....  .. . :..:. .:  ..:..::: . ::. .   :.:: : ..
ABC1K3 ALNYTVSELNFKTLVDGLGAVFYQYPFNVPPYYALILRSLTVLEGLALYADPNFKVLAAS
        460       470       480       490       500       510

       540       550       560       570       580
ABC1K1 YPYVAKRLLTDPNPALRERLIQVLFKDGVFQWKRLENLLSLAKEN-----------VAKM
       ::: :::::::::: ::. ::..::::: :.:.::::::. ....           : :.
ABC1K3 YPYFAKRLLTDPNPYLRDALIELLFKDGKFRWNRLENLLQQGSKDRDFSAKDALQPVLKL
        520       530       540       550       560       570

        590          600       610                   620       630
ABC1K1 SSNPN---LRVKRVESKLDLTDTIKDGA------------RLFLLDEGIRRKLILALTE-
         .::   ::.  ..  . ....:  :.            : .... .    : ..  .:
ABC1K3 LLDPNGEELRLLVIKEAVRVSEAIALGTVVDTYNSLPEFLRSLVFNGNGNGPLTMSTAEL
        580       590       600       610       620       630

              640        650        660
ABC1K1 DSKLHVEELVD-VYRLVEDEVDI-PTLAMQVVQDL--PNVFR------------------
       .: :.... :. .. :...  .. :.. . ..: :  :.. :
ABC1K3 QSTLELRDQVSRIWGLLQSSESFDPAILQPILQVLQQPEARRLGGRVAGGVGQRLAARFL
        640       650       660       670       680       690

       670       680
ABC1K1 -DFVLSWSNSVLSDR
        ... . . :   .
ABC1K3 QQLLRATTPSSAPSP
        700       710
```

Global alignment
31.6% identity
(61.4% similar)

Protein kinase domain
IPR000719
42.3% identity
(74.8% similar)

UbiB domain
IPR004147
44.2% identity
(73.6% similar)

Supplementary Figure 1. Alignment between ABC1K1 and ABC1K3 protein sequences. Result of the alignment between the two protein sequences performed with lalign program (https://embnet.vital-it.ch/software/LALIGN_form.html). The predicted kinase domain is highlighted in green, the predicted UbiB domain in bold and underlined. The box shows the identity and similarity scores for the global/global, kinase domains and UbiB alignments.

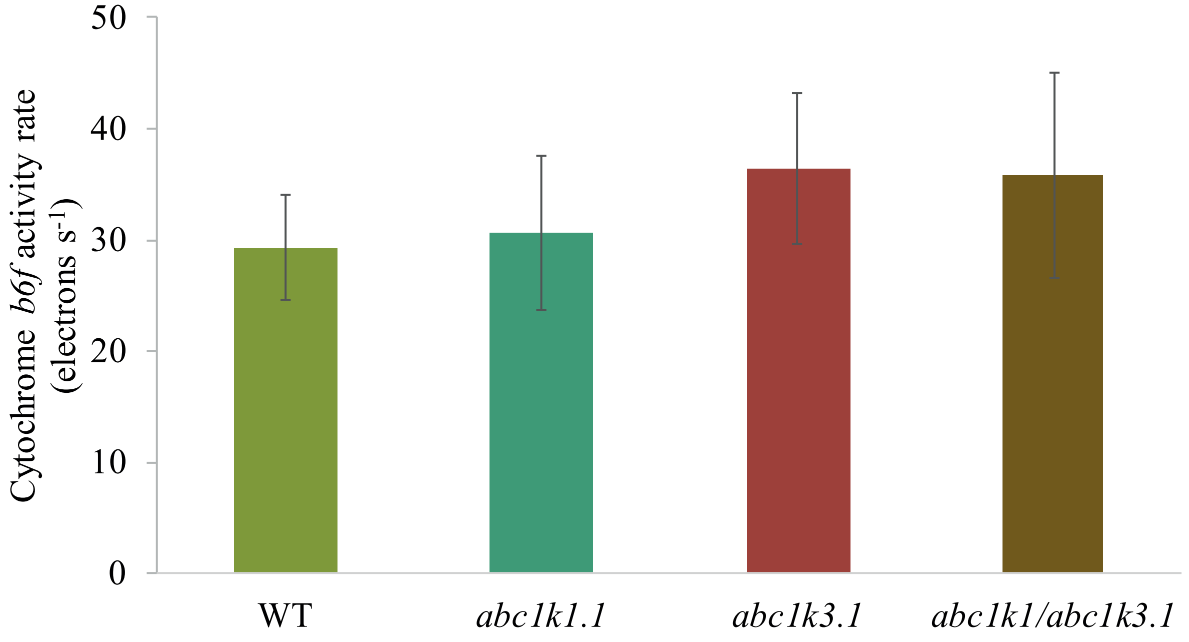

Supplementary Figure 2. Cytochrome *b6f* activity rate is not affected by *abc1k1* and/or *abc1k3* mutation. Turnover rate of cytochrome *b6f* , expressed as number of electrons per second, calculated from the kinetics of the cytochrome *f* oxidation after a saturating pulse fitted with an exponential curve. The kinetics were obtained from the changes in the cytochrome *f* absorption at 554 nm as described in Finazzi *et al.* (2002). The error bars show the standard deviation of the points (n=4).

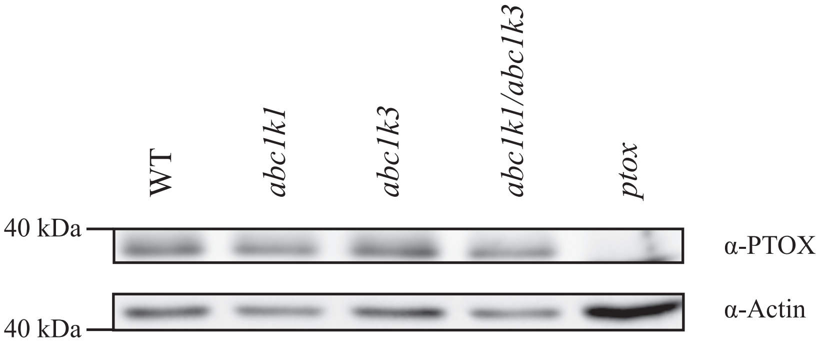

Supplementary Figure 3. Accumulation of PTOX enzyme. Total leaf protein extracts from 4 weeks-old wild type (WT), *abc1k1-1, abc1k3-1, abc1k1/abc1k3-1* and *ptox* plants grown under moderate light, analysed by immunoblotting with anti-PTOX antibody. Actin was used as a loading control.

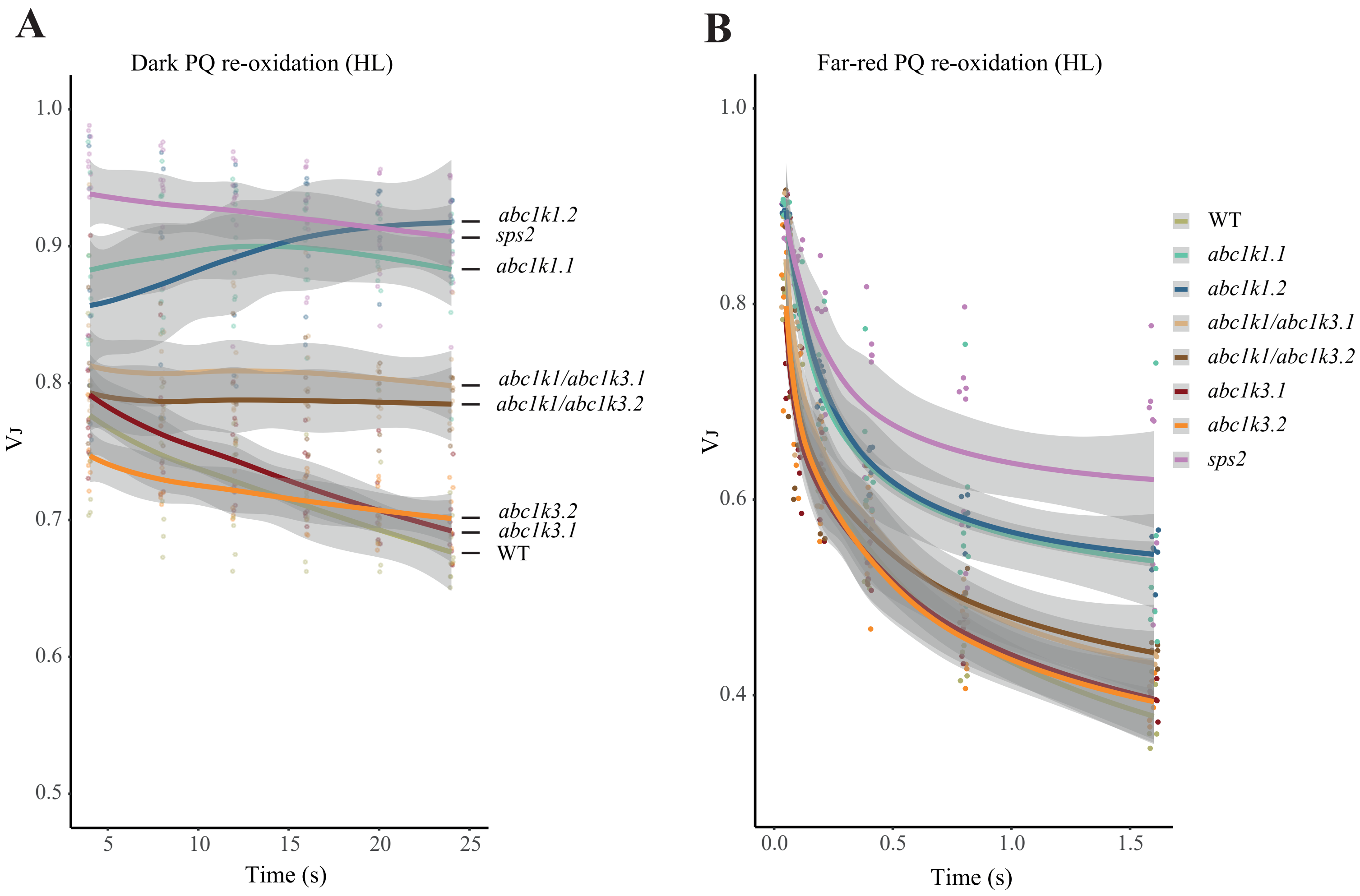

Supplementary Figure 4. Enzymatic dark re-oxidation PQ after high light. Time course of fluorescence quenching kinetics during incubation in dark (**A**) or after exposure to increasing time with far-red (**B**) of wild type (WT), *abc1k1.1, -2*, *abc1k3.1, -2* and *abc1k1/abc1k3.1, -2*, *sps2*, *stn7/stn8*, *ptox* plants. Before leaf collection the plants were exposed for 3 hours under high light (500 µmol m^{-2} s^{-1}) (HL). The values of the VJ were plotted over the time after the previous saturating flash and interpolated with a logarithmic curve (R-Studio). The measures were performed with Handy-PEA (Hansatech Instruments).

Genes	Primer Names	Sequences
abc1k1.1	LP1.1	AGC TGA TTC ATC ATC TGT CGG
	RP1.1	TCC CTT CCC ACA CTA AAA GT
abc1k3.1	LP3.1	TGT TGC TGT CAA AGT TCA ACG
	RP3.1	CAA GCG TAC TTT GAA GTT CCG
abc1k3.2	LP3.2	GGG AGG AGG TAG TGA CAA AGG
	RP3.2	AAG GTA ATC GGG TGG ACA GAG
SALK TDNA	LBb1.3	ATT TTG CCG ATT TCG GAA C
SAIL TDNA	LB3_Sail	TAG CAT CTG AAT TTC ATA ACC AAT CTC GAT ACA C

Supplementary Table 1. Primers sequences for genotyping of *abc1k1/abc1k3* double murant. Left-primers (LP) and right-primers (RP) used for ABC1K1 (1.1) or ABC1K3 (3.1 or 3.2) genes. LBb1.3 and LB3_Sail primers were used for Salk and Sail TDNA insertions respectively.
